# Genomic insights into multidrug and extensively drug-resistant *Klebsiella pneumoniae* from bloodstream infections at three Zambian tertiary hospitals

**DOI:** 10.3389/frabi.2026.1933211

**Published:** 2026-10-09

**Authors:** Joseph Yamweka Chizimu, Harvey Kakoma Kamboyi, Maisa Kasanga, Taona Sinyawa, Misheck Shawa, Ruth Nakazwe, Steward Mudenda, Joseph Njung’e, Susan Kiiru, Mungunsar Chuluun, Chileshe Lukwesa, Zoran Muhimba, Victor Daka, Dianah Tibatemwa, Everlyn Wesangula, O-Tipo Shikanga, Sandra Diana Mwadetsa, Nchimunya Machila, Veronica Chuchu, Zachariah Mupila, Peter Clement Lasuba, Yasuhiko Suzuki, Flavien Nsoni Bumbangi, Roma Chilengi

**Affiliations:** 1 Antimicrobial Resistance Coordinating Committee, Zambia National Public Health Institute, Ministry of Health, Lusaka, Zambia; 2 Directorate of Research, Eden University, Lusaka, Zambia; 3 Department of Medicine and Clinical Sciences, School of Medicine, Eden University, Lusaka, Zambia; 4 Central Veterinary Research Institute, Ministry of Fisheries and Livestock, Lusaka, Zambia; 5 Hokudai Center for Zoonosis Control in Zambia, Hokkaido University, Lusaka, Zambia; 6 Division of Infection and Immunity, International Institute for Zoonosis Control, Hokkaido University, Sapporo, Japan; 7 Department of Pathology and Microbiology, University Teaching Hospital, Lusaka, Zambia; 8 Department of Pharmacy, School of Health Sciences, University of Zambia, Lusaka, Zambia; 9 Digital Health, Commonwealth Pharmacists Association, Kampala, Uganda; 10 Centre for Microbiology Research, Kenya Medical Research Institute, Nairobi, Kenya; 11 Institute of Basic and Biomedical Sciences, Levy Mwanawasa Medical University, Lusaka, Zambia; 12 Department of Pathology, University Teaching Hospitals, Ministry of Health, Lusaka, Zambia; 13 Public Health Department, School of Medicine, Copperbelt University, Ndola, Zambia; 14 Surveillance and Prescribing Support for Antimicrobial Stewardship Resource Capacity Building, Commonwealth Pharmacists Association, Kampala, Uganda; 15 East, Central and Southern Africa Health Community, Arusha, Tanzania; 16 Department of Health, World Health Organisation, Lusaka, Zambia; 17 Commonwealth Pharmacists Association, London, United Kingdom; 18 Division of Bioresources, Hokkaido University International Institute for Zoonosis Control, Sapporo, Japan; 19 Division of Research Support, Hokkaido University Institute for Vaccine Research and Development, Sapporo, Japan

**Keywords:** antimicrobial resistance (AMR), high-risk clones, *Klebsiella pneumoniae*, MDR - (multi drug resistant), XDR (extensively drug resistant), Zambia

## Abstract

**Background:**

*Klebsiella pneumoniae* is a leading cause of hospital-associated infections and a major contributor to antimicrobial resistance (AMR) worldwide. Despite its importance and prioritisation in Zambia’s National Action Plan on AMR, genomic surveillance information in sub-Saharan Africa remains limited. This study investigated the population structure, determinants of AMR, and plasmid diversity of *K. pneumoniae* isolates from three tertiary hospitals in Zambia.

**Methods:**

Thirty archived *K. pneumoniae* blood isolates collected between December 2023 and May 2025 from University Teaching Hospitals (UTH), Ndola University Teaching Hospital (NUTH), and Livingstone University Teaching Hospital (LUTH) and subjected to antimicrobial susceptibility testing (AST) and whole-genome sequencing (Illumina NextSeq 2000). Sequence types (STs), plasmid replicons, and antimicrobial resistance genes (ARGs) were identified using MLST, PlasmidFinder, and the Comprehensive Antibiotic Resistance Database, respectively.

**Results:**

Twenty-eight isolates (93%) were multidrug-resistant, of which seven (25%) were extensively drug-resistant (XDR), exhibiting resistance across eight antibiotic classes, including carbapenems. Whole-genome sequencing identified 18 sequence types (STs), including two novel STs, and globally disseminated high-risk clones such as ST11, ST15, ST101, ST307, ST377, and ST607, which are circulating across hospitals. UTH exhibited the highest diversity of STs. The seven XDR *K. pneumoniae* isolates, six (85%) were from UTH, while the remaining isolate was from NUTH. Among the XDR isolates, five belonged to clinically dominant clones, comprising ST377 (two isolates) and single representatives of ST101, ST307, and ST607. The other two were ST13 and ST831, which have not been reported to have an XDR profile. All isolates carried multiple ARGs, dominated by those encoding β-lactamases (*bla*_CTX-M-15_: 93%, *bla*_TEM_: 70%), aminoglycoside-modifying enzymes (*aac(6’)-Ib-cr5*: 77%, *aac(3)-IIe*: 64%), and efflux pump genes (*emrD*: 100%). The carbapenemase-encoding gene, *bla*_NDM-5_, was present in 13% of the isolates.

**Conclusions:**

This first genomic survey of *K. pneumoniae* in Zambia reveals extensive clonal diversity dominated by global high-risk lineages carrying plasmid-mediated ESBL and carbapenemase genes. The detection of XDR strains, particularly at UTH, underscores the urgent need to strengthen laboratory surveillance across all tertiary hospitals in Zambia and improve infection prevention programmes.

## Introduction

*Klebsiella pneumoniae* is a Gram-negative opportunistic pathogen of global concern, frequently implicated in healthcare-associated infections, including pneumonia, urinary tract infections, surgical-site infections, and bloodstream infections (Gorrie et al., 2022). *K. pneumoniae* is a prominent member of the *K. pneumoniae* species complex that comprises five closely related species distributed across seven major phylogroups, including *K. pneumoniae, K. quasipneumoniae, K. variicola, K. quasivariicola*, and *K. Africana* (Rodrigues et al., 2018; Barbier et al., 2020). *K. oxytoca* is not a member of the *K. pneumoniae* species complex but belongs to the separate *K. oxytoca* species complex (Yang et al., 2022). *K. pneumoniae* is a common opportunistic hospital-associated pathogen, accounting for about one-third of all Gram-negative infections overall (Chung, 2016). Alongside its high prevalence, *K. pneumoniae* is a major source of antimicrobial resistance (AMR). It is evident that AMR rates in *K. pneumoniae* have been steadily increasing over the years (Chung, 2016; Jalal et al., 2023; WHO, 2024). Resistance levels vary considerably between countries, largely driven by differences in the prevalence of extended-spectrum β-lactamase (ESBL) production, with non-susceptibility rates exceeding 50%–60% for third-generation cephalosporins, fluoroquinolones, and aminoglycosides (Chung, 2016). In sub-Saharan Africa (SSA), meta-analyses and systematic reviews indicate that resistance to first-line agents in *K. pneumoniae* often exceeds 50% across many settings (Kariuki et al., 2022; Gebremeskel et al., 2023; Kijineh et al., 2024). A recent study in Zambia found that *K. pneumoniae* accounts for a significant burden of AMR and clinical disease, comprising approximately 30% of ESBL-producing Gram-negative bloodstream infections, with 88% resistant to cefootaxime, 77% to ceftazidime, and 62% to ciprofloxacin (Yamba et al., 2023). Further, multidrug-resistant (MDR) and extensively drug-resistant (XDR) *K. pneumoniae* are increasingly reported across sub-Saharan Africa. Studies from South Africa, Nigeria, and Tanzania document the spread of high-risk clones, such as ST15, ST101, and ST307, that carry ESBL and carbapenemase genes, including *bla*_CTX-M-15_, *bla*_KPC_, and *bla*_NDM_ (Asare Yeboah et al., 2025; Hetsa et al., 2025; Olaitan et al., 2025).

Its ability to acquire and disseminate antimicrobial-resistance genes (ARGs) and virulence determinants via plasmids and other mobile genetic elements renders it a formidable threat, particularly in resource-limited settings (Wyres et al., 2020). The accessory genome of *K. pneumoniae* is central to its success as a nosocomial pathogen; the plasmid-borne ARGs and virulence loci converge into so-called “high-risk clones” that pose severe treatment challenges (Abdelsalam et al., 2024; Huang et al., 2025). From a genomic standpoint, Multilocus Sequence Typing (MLST) and Whole-Genome Sequencing (WGS) have advanced the understanding of *K. pneumoniae* epidemiology, revealing global clone lineages such as ST11, ST15, ST147, ST258 and ST307, which harbour extensive ARG repertoires and are associated with outbreaks (Peirano et al., 2020; Shaidullina et al., 2023).

Despite this global threat, genomic surveillance of *K. pneumoniae* in many sub-Saharan African countries remains limited (Cornick et al., 2021; Aruhomukama and Nakabuye, 2023; Verani et al., 2024). In Zambia, as in many low- and middle-income countries (LMICs), the AMR situation is recognised as a One Health crisis, driven by high rates of non-prescribed antimicrobial use (AMU) in humans and animals, coupled with limited diagnostic capacity and weak regulatory enforcement, posing a significant threat to public health and food security (Mudenda et al., 2025). Concerted national action, guided by the Multisectoral National Action Plan on AMR, is focused on strengthening surveillance, stewardship, and public awareness to curb its spread (ZNPHI, 2017). However, genomic capacity is constrained (Sinyawa et al., 2025), thus, there is a critical gap in our understanding of *K. pneumoniae* diversity, transmission dynamics, ARG, and virulence gene carriage in Zambian hospitals (Inzaule et al., 2021; Heinz et al., 2024; Shawa et al., 2025).

Here, we present the first genomic surveillance of *K. pneumoniae* from three hospitals in Zambia: the national referral University Teaching Hospital (UTH) in Lusaka Province, the tertiary Ndola University Teaching Hospital (NUTH) in the Copperbelt Province, and Livingstone University Teaching Hospital (LUTH) in the Southern Province. Using WGS, we characterised the population structure, plasmid replicons, ARGs, and compared across hospitals. We aimed to map the genomic epidemiology of *K. pneumoniae* across Zambian hospital settings and to provide baseline data to inform antimicrobial stewardship (AMS) and infection prevention and control (IPC) interventions.

## Methods

### Study design and sample collection

A cross-sectional study was conducted on clinical isolates of *K. pneumoniae* collected between December 2023 and May 2025 from three hospitals in Zambia, UTH, NUTH, and LUTH. Clinical isolates of *K. pneumoniae* were obtained from blood samples collected for routine laboratory examination from patients admitted to the above tertiary hospitals. A total of 32 presumptively identified *K. pneumoniae* were collected and biobanked during this period, but only 30 (UTH: *n* = 21, NUTH: *n* = 6, and LUTH: *n* = 3) were sequenced. Two isolates were excluded due to insufficient DNA quality for sequencing. Although *K. pneumoniae* is the leading cause of bacteraemia in patients, isolation rates from blood cultures are very low (Inzaule et al., 2021; Clinical and Laboratory Standards Institute, 2023; Heinz et al., 2024; Shawa et al., 2025), notwithstanding resource constraints in sample collection and processing.

### Species identification and antimicrobial susceptibility testing

Species identification and AST were conducted using the VITEK Compact 2 automated system (bioMérieux, France) at the Zambia National Public Health Institute Reference Laboratory in Lusaka. Prior to use, the VITEK 2 system was subjected to routine quality control using the reference strain, *E. coli* ATCC25922, in accordance with the manufacturer’s recommendations. Isolates were suspended in sterile saline to achieve a turbidity of 0.5-0.63 McFarland standard. VITEK 2 Gram-negative (GN) identification cards and AST-GN81 cards were inoculated according to the manufacturer’s instructions. The AST panel consisted of penicillins (ampicillin, amoxicillin–clavulanic acid, piperacillin–tazobactam), cephalosporins (cefazolin, cefoxitin, ceftazidime, ceftriaxone, and cefepime), carbapenems (ertapenem and meropenem), aminoglycosides (amikacin, gentamicin and tobramycin), fluoroquinolones (ciprofloxacin and levofloxacin), tetracycline, and folate pathway inhibitors (trimethoprim-sulfamethoxazole). The minimum inhibitory concentration (MIC) was interpreted as susceptible, intermediate, or resistant according to the CLSI M100-S34 guidelines (Abdelsalam et al., 2024).

### Whole-genome sequencing and analyses

Genomic DNA was extracted from overnight cultures using the DNeasy Blood and Tissue Kit (Qiagen, Germany) following the manufacturer’s protocol for GN bacteria. Libraries were prepared using the Nextera XT DNA Library Preparation Kit (Illumina, USA) according to the manufacturer’s instructions. Pooled libraries were denatured with 0.2 N NaOH and diluted to a final loading concentration of 1.8 pM with 1% PhiX control (Illumina, USA). Sequencing was performed on the Illumina NextSeq 2000 platform using the NextSeq 2000 Mid Output Kit v2.5 (300 cycles), generating 150 bp paired-end reads. Raw sequencing reads were assessed for quality using FastQC v0.11.9 (Andrews, 2010). Adapter sequences and low-quality bases (Phred score <20) were trimmed using Trimmomatic v0.39 (Bolger et al., 2014) with default parameters. High-quality reads were *de novo* assembled using SPAdes v3.15.3 (Bankevich et al., 2012) with the “--careful” option and k-mer sizes of 21, 33, 55, 77, 99, and 127. Assembly quality metrics, including N50, L50, total assembly length, and number of contigs, were evaluated using QUAST v5.0.2 (Gurevich et al., 2013). Assemblies with N50 values >50,000 bp and <300 contigs were considered high quality and retained for downstream analysis.

Phylogenetic analysis was performed using assembled contig fasta files and RefSeq fasta files of complete genomes of *K. pneumoniae* (*n* = 60) downloaded from NCBI. In addition, RefSeq genomes of *K. quasipneumoniae* (*n* = 3), *K. variicola* (*n* = 5), and *K. oxytoca* (*n* = 6) were included for quality control within the *K. pneumoniae* species complex. Single-nucleotide variants were identified by mapping each genome against the *K. pneumoniae* HS11286 reference genome using Snippy v4.6.0 (Seemann). A core-genome alignment was generated using Snippy-core v4.6.0 (Seemann), and a maximum-likelihood phylogenetic tree was inferred from the resulting core alignment using IQ-TREE 2 v3.1.3 (Minh et al., 2020), employing ModelFinder (Kalyaanamoorthy et al., 2017) to determine the best-fit substitution model and 1,000 ultrafast bootstrap replicates. The resulting phylogenetic tree was visualised and annotated using FigTree v1.4.4 (Rambaut).

### Species confirmation and multilocus sequence typing

Species identification was confirmed in silico using Kraken2 v2.1.2 (Wood et al., 2019) and SpeciesFinder (Larsen et al., 2014) against the respective RefSeq bacterial database. Multilocus sequence typing (MLST) was performed using MLST v2.19.0 (Seemann, 2015) with the *K. pneumoniae* MLST scheme from the PubMLST database (https://pubmlst.org/). Sequence types (STs) were assigned based on allelic profiles of seven housekeeping genes: *gapA, infB, mdh, pgi, phoE, rpoB*, and *tonB*.

### AMR and virulence genes detection and plasmid characterisation

AMR genes were identified using the Comprehensive Antibiotic Resistance Database (CARD) v3.2.8 with the Resistance Gene Identifier (RGI) v5.2.1 (Alcock et al., 2019) using “perfect” and “strict” hit criteria. Additional AMR gene screening was performed using ResFinder v4.1 (Bortolaia et al., 2020) with a minimum identity threshold of 90% and a minimum coverage of 60%. Detected ARGs were categorised by antimicrobial class.

Virulence genes were identified from assembled *K. pneumoniae* genomes using ABRicate v0.5 against the VFDB database (Chen, 2004), with thresholds of ≥90% sequence coverage and ≥90% nucleotide identity. Results were summarised across all 30 isolates, and an AWK script (Aho et al., 1979) converted gene detection into a binary presence/absence matrix (1 = present; 0 = absent).

Plasmid replicons were identified using PlasmidFinder v2.1 (Carattoli et al., 2014) with default parameters (minimum identity 95%, minimum coverage 60%). Plasmids were classified according to their incompatibility (Inc) groups. Plasmid MLST (pMLST) was performed for IncF plasmids using pMLST v2.0 (Carattoli and Hasman, 2020).

### Data visualisation and analysis

Data visualisation was performed using GraphPrism 8.4.3. Heatmaps were generated to visualise the AST results of the isolates and the presence/absence of virulence genes and ARGs across sequence types. Descriptive statistics were performed using GraphPad Prism 8.4.3 to summarise the frequencies and prevalence of genetic determinants. The AST results and AMR gene presence shown in a dendrogram were constructed using R version 2025.05.1 + 513. The tidyverse packages were used to prepare and organise the data. The Circlize package was used to define colour mapping function and ComplexHeatmap package was used for hierarchical clustering and dendrogram construction.

### Ethical considerations

Ethical clearance was obtained from the Tropical Diseases Research Centre Ethics Committee under protocol ID TRC/C4/09/2023. Approval to conduct and implement this study was also obtained from the Zambia National Health Research Authority (NHRA) and the management of the Zambia National Public Health Institute (ZNPHI). This study involved the analysis of de-identified, routine laboratory data, with no direct patient contact, and therefore posed minimal risk to participants.

## Results

### Antimicrobial resistance profiles and patterns

Antimicrobial susceptibility tests identified high levels of non-susceptibility to multiple drug classes, including carbapenems. High non-susceptibility was also observed among penicillin/β-lactamase inhibitor combinations, {[[amoxicillin–clavulanic acid (63%, *n* = 19) and piperacillin–tazobactam (40%, *n* = 12)}, cefazolin (93%, *n* = 28), ceftazidime (87%, *n* = 26), ceftriaxone (90%, *n* = 27), and cefepime (90%, *n* = 27), gentamicin (60%, *n* = 18), tobramycin (57%, *n* = 17), ciprofloxacin (47%, *n* = 14), levofloxacin (37%, *n* = 11), tetracycline (60%, *n* = 18), and trimethoprim-sulfamethoxazole (90%, *n* = 27) (**Figure 1A**). High levels of resistance to penicillin/β-lactamase inhibitor combinations, first, third and fourth-generation cephalosporins indicates a likelihood of ESBL production among the majority of isolates. Moderate non-susceptibility to cefoxitin (27%, *n* = 8), ertapenem (23%, *n* = 7), and meropenem (17%, *n* = 5) was observed. All isolates were susceptible to amikacin (**Figure 1A**). Twenty-eight (28, 93%) isolates were classified as MDR, i.e., resistant to three or more drug classes except for ZLU0042269 and ZLU0042373, which were resistant to one antibiotic class. Seven (25%) of the MDR isolates were XDR, showing non-susceptibility to multiple antimicrobial classes, including carbapenems, but were susceptible only to amikacin among the aminoglycosides (**Figure 1B**).

**Figure 1:**
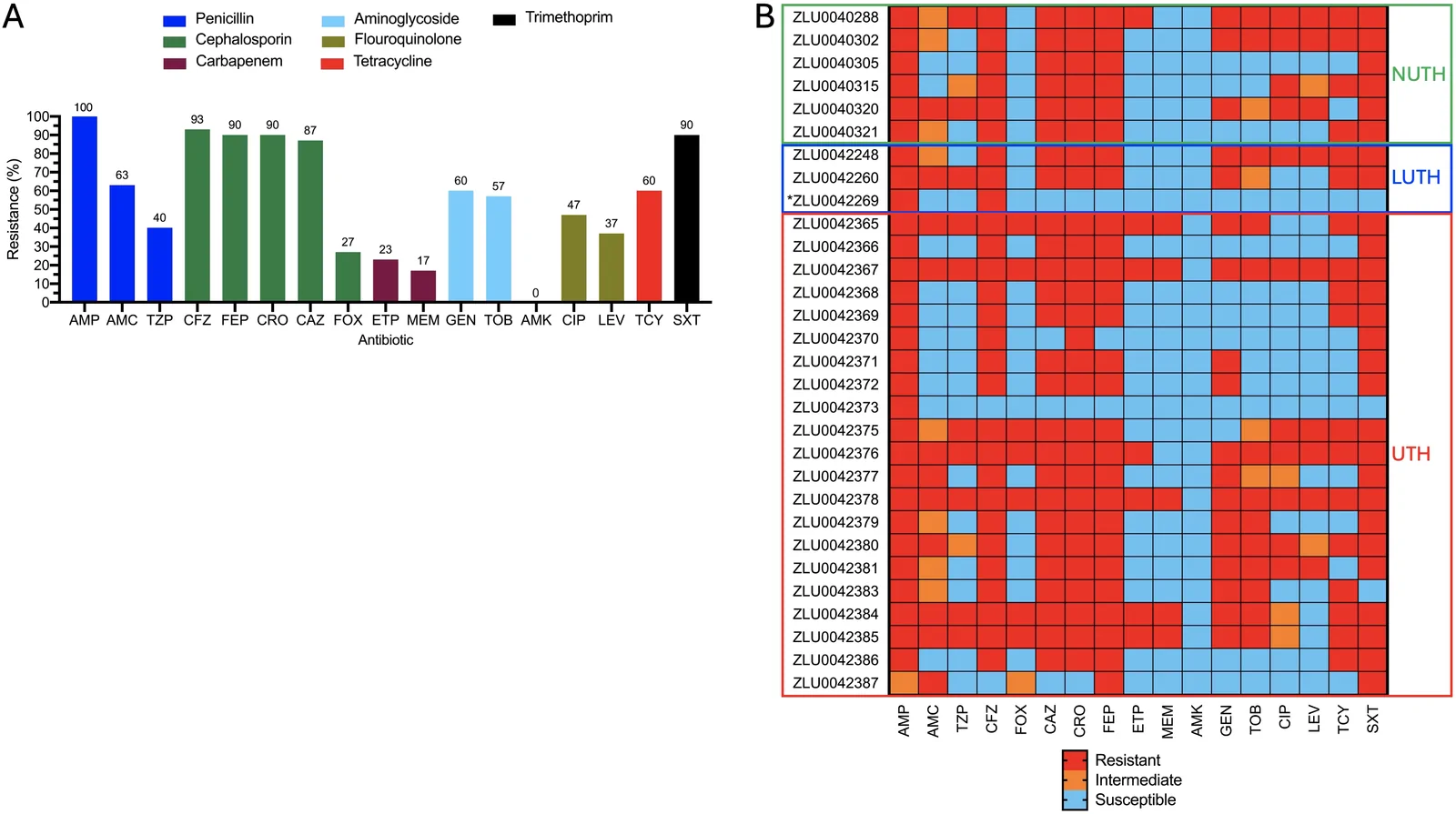
Antimicrobial resistance patterns of 30 *K. pneumoniae* as determined by the VITEK 2 Compact automated system. **(A)** Antimicrobial resistance rates of the *K. pneumoniae* to 17 antibiotics representing 7 antibiotic classes. **(B)** Heatmap of antimicrobial susceptibility patterns of the *K. pneumoniae* isolates from NUTH, LUTH, and UTH. AMP, ampicillin; AMC, amoxicillin–clavulanic acid; TZP, piperacillin–tazobactam; CFZ, cefazolin; FOX, cefoxitin; CAZ, ceftazidime; CRO, ceftriaxone; FEP, cefepime; ETP, ertapenem; MEM, meropenem; AMK, amikacin; GEN, gentamicin; TOB, tobramycin; CIP, ciprofloxacin; LEV, levofloxacin; TCY, tetracycline; SXT, trimethoprim-sulfamethoxazole; *K. oxytoca.

### Diverse sequence types within and among three tertiary hospitals

Species identification of the 30 presumptively identified *K. pneumoniae* isolates confirmed *K. pneumoniae* as the predominant species. Of the 30 sequenced isolates, 29 (96.7%) were identified as *K. pneumoniae*, while one isolate (ZLU0042269) was identified as *K. oxytoca*. MLST of the 29 *K. pneumoniae* identified 18 distinct sequence types (STs) and 2 novel STs designated Novel1and Novel2. The distinct STs included five dominant hospital-associated STs, ST11 (*n* = 1), ST15 (*n* = 2), ST307 (*n* = 3), ST377 (*n* = 2) and ST607 (*n* = 2), as well as several minor STs (*n* = 13) (**Figure 2**). The *K. pneumoniae* ST Novel1 had uncertain alleles *gapA*(326)?, *infB*(256)?, and pgi(*399*)?, while Novel2 had a complete allelic profile, but MLST assigned no ST (**Supplementary Table S1**).

**Figure 2:**
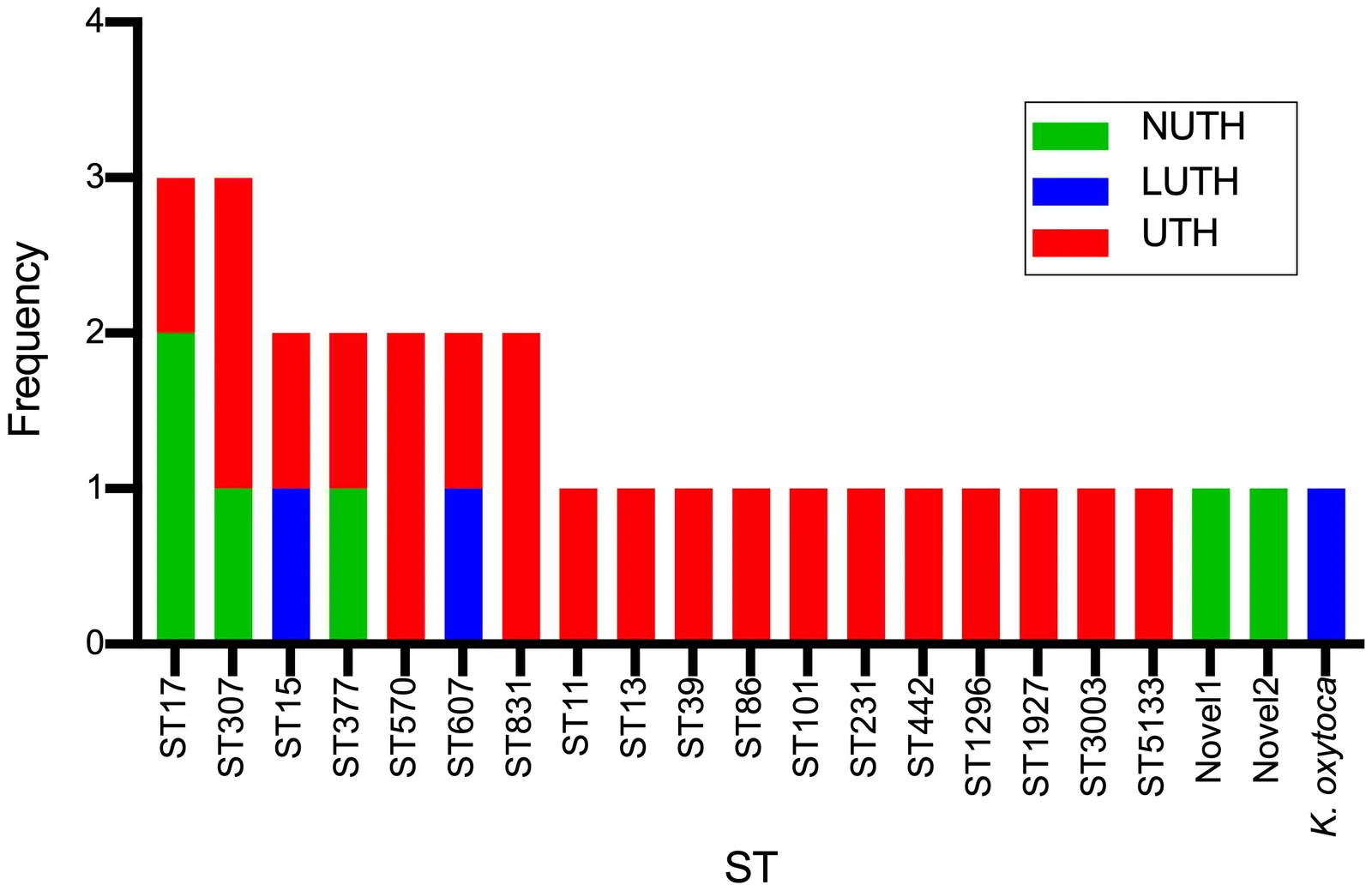
*K. pneumoniae* STs identified in this study. Frequency of the STs among the 30 isolates and the distribution of *K. pneumoniae* STs per tertiary hospital.

Distribution of isolates across hospitals was NUTH (20%, *n* = 6), LUTH (10%, *n* = 3), and UTH (70%, *n* = 21). UTH had a diverse distribution of STs, possessing all five high-risk *K. pneumoniae* clones: ST11, ST15, ST307, ST377, and ST607. While NUTH had ST307 and 377, LUTH possessed ST15 and ST607 (**Figure 2**).

### STs co-carried multiple plasmid replicons dominated by IncFIB(K)_1_Kpn3

Nine plasmid replicons were identified, with IncFIB and IncFII being dominant, followed by IncR and Col-type plasmids across all STs in this study. The IncFIB(K)_1_Kpn3 plasmid replicon was by far the most predominant plasmid replicon (*n* = 26) among the STs. It is known to be associated with 51 different resistance profiles, including ESBLs and carbapenemases (Hetsa et al., 2025) (**Figure 3A**). It was followed by IncFII_1_pKP91 (*n* = 16), while the IncHI1B_1_pNDM-MAR plasmid replicon, related to the pNDM-MAR prototype, was present in 6 isolates. The IncHI1B_1_pNDM-MAR plasmid replicon is strongly associated with NDM-mediated carbapenem resistance as well as MDR or even XDR phenotypes (Ragupathi et al., 2019; Shapovalova et al., 2024).

**Figure 3:**
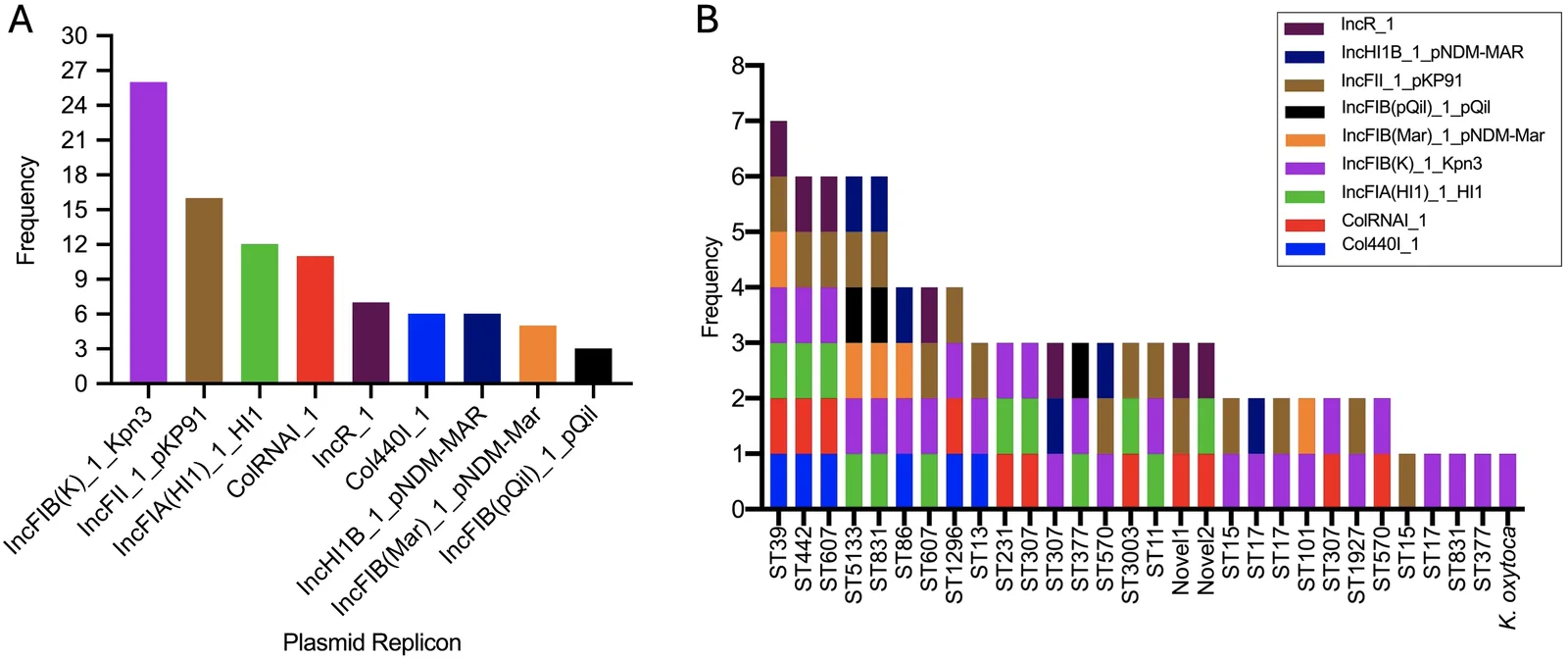
Multiple plasmid replicons with co-carriage among the isolates. **(A)** The IncFIB(K)_1_Kpn3 plasmid replicon was the most common replicon found in 86% (*n* = 26). **(B)** More than 83% (25/30) of the isolates co-carried plasmid replicons.

Co-carriage of multiple replicons was frequent, particularly in ST39, ST442, ST607, ST5133, ST831, ST86 and ST1296 had at least 4 plasmid replicons, all bearing the IncFIB(K)_1_Kpn3 plasmid replicon (**Figure 3B**). Only ST15, ST3003, Novel1 and Novel2 did not possess the IncFIB(K)_1_Kpn3 replicon (**Figure 3B**). Interestingly, non-high-risk clones of *K. pneumoniae* ST39, ST86, ST101, ST5133, and ST831 co-harboured the IncFIB(K)_1_Kpn3 and IncHI1B_1_pNDM-MAR replicons.

### ARG profiles across sequence types

Analysis of the ARGs against each ST showed that all STs in this study harboured multiple ARGs. ST5133 (ZLU0042373) and *K. oxytoca* (ZLU0042269) had the fewest ARGs. The ST5133 (ZLU0042373) carried four ARGs (*bla*_SHV-11_, *emrD*, *fosA*, and *oqxA*), whereas *K. oxytoca* (ZLU0042269) carried only two (*emrD* and *oqxA*). The rest harboured at least 11 ARGs, with ST13 and ST442 carrying 25 of the 40 ARGs identified in this study (**Figure 4**). Other clinically significant and dominant STs (“high-risk clones”), such as ST11, ST15, ST101, ST307, ST377, and ST607, harboured up to 13, 17, 16, 21, 13, and 18 ARGs, respectively, with potential for MDR (**Figure 4**).

**Figure 4:**
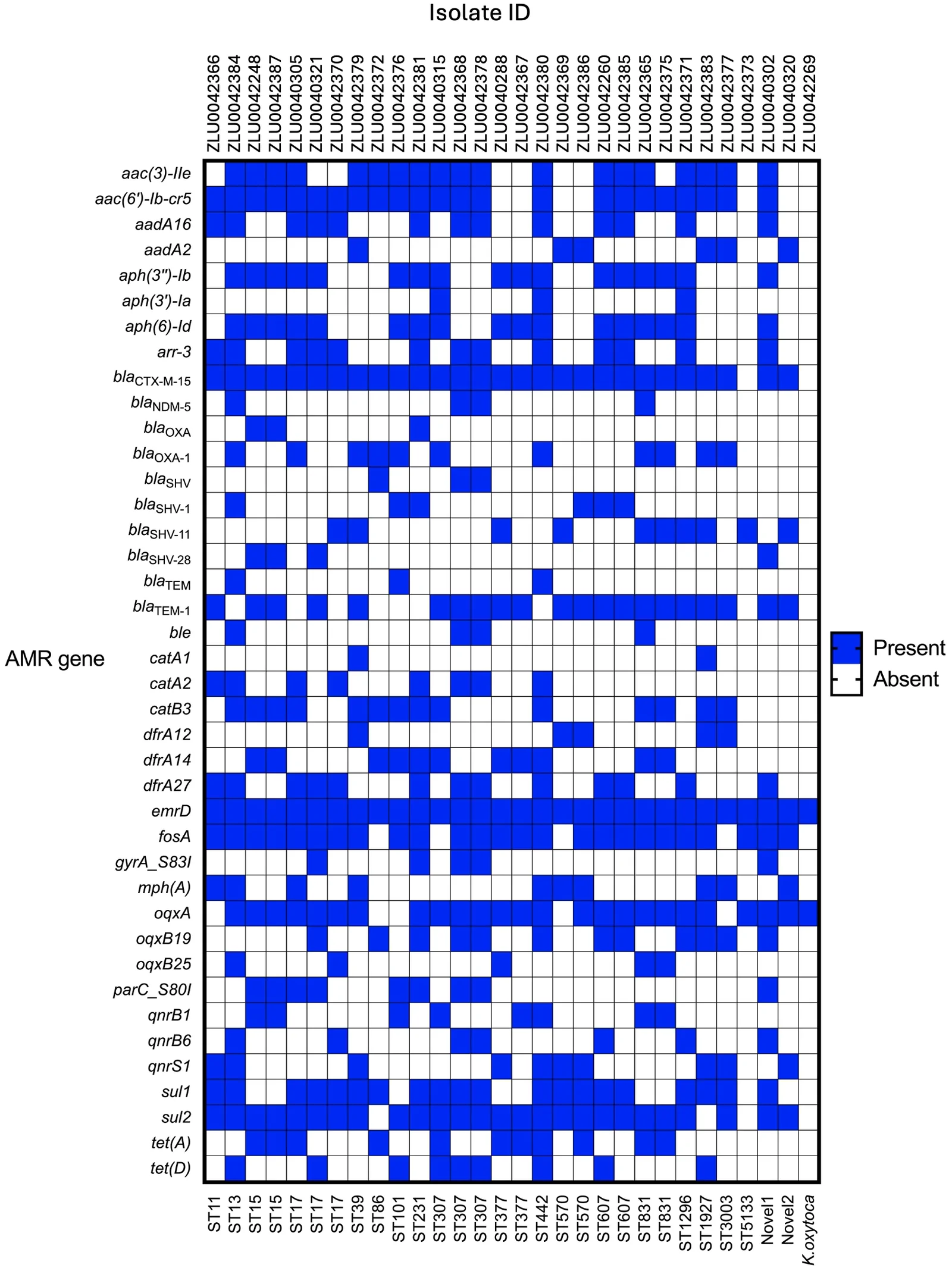
Heatmap of ARGs across isolates showing the dominance of genes encoding aminoglycoside-modifying enzymes (*aac(3)-IIe* and *aac(6′)-Ib-cr5*), ESBL (*bla*_CTX-M-15_ and *bla*_TEM_), multidrug efflux protein (*emrD*), fosfomycin resistance (*fosA*), and sulfonamide resistance (*sul1* and *sul2*).

Further, the frequency of ARGs was dominated by β-lactamases (*bla*_CTX-M-15_ in 93%, *bla*_TEM_ in 70%), while carbapenemase-encoding *bla*_NDM-5_ was detected in 13% of isolates (**Figure 5**). The most prevalent aminoglycoside resistance gene was *aac(6’)-Ib-cr5* (77%), followed by *aac(3)-IIe* (63%), *aph(3’)-Ib* (57%), *aph(6)-Id* (57%) and *aadA16* (43%). Further, all isolates carried *emrD*, a gene encoding a multidrug efflux protein involved in adaptation to low-energy shock (Naroditskaya et al., 1993). The fosfomycin resistance gene (*fosA*) was also present in 25 isolates (83%). Quinolone resistance genes were also highly prevalent, with *oqxA* (83%) being the most common, followed by *oqxB19* (40%). The dual aminoglycoside/quinolone resistance conferred by *aac(6’)-Ib-cr5* (77%) further increases the prevalence of quinolone ARGs. Sulfonamide resistance genes were also common with *sul1* and *sul2* at 67% and 87%, respectively. Finally, *catB3-encoding* resistance to phenicols had a prevalence of 47%. The remaining genes conferring resistance to glycopeptides, macrolides, fluoroquinolones, and tetracyclines were present at relatively lower rates (<40%) (**Figure 5**).

**Figure 5:**
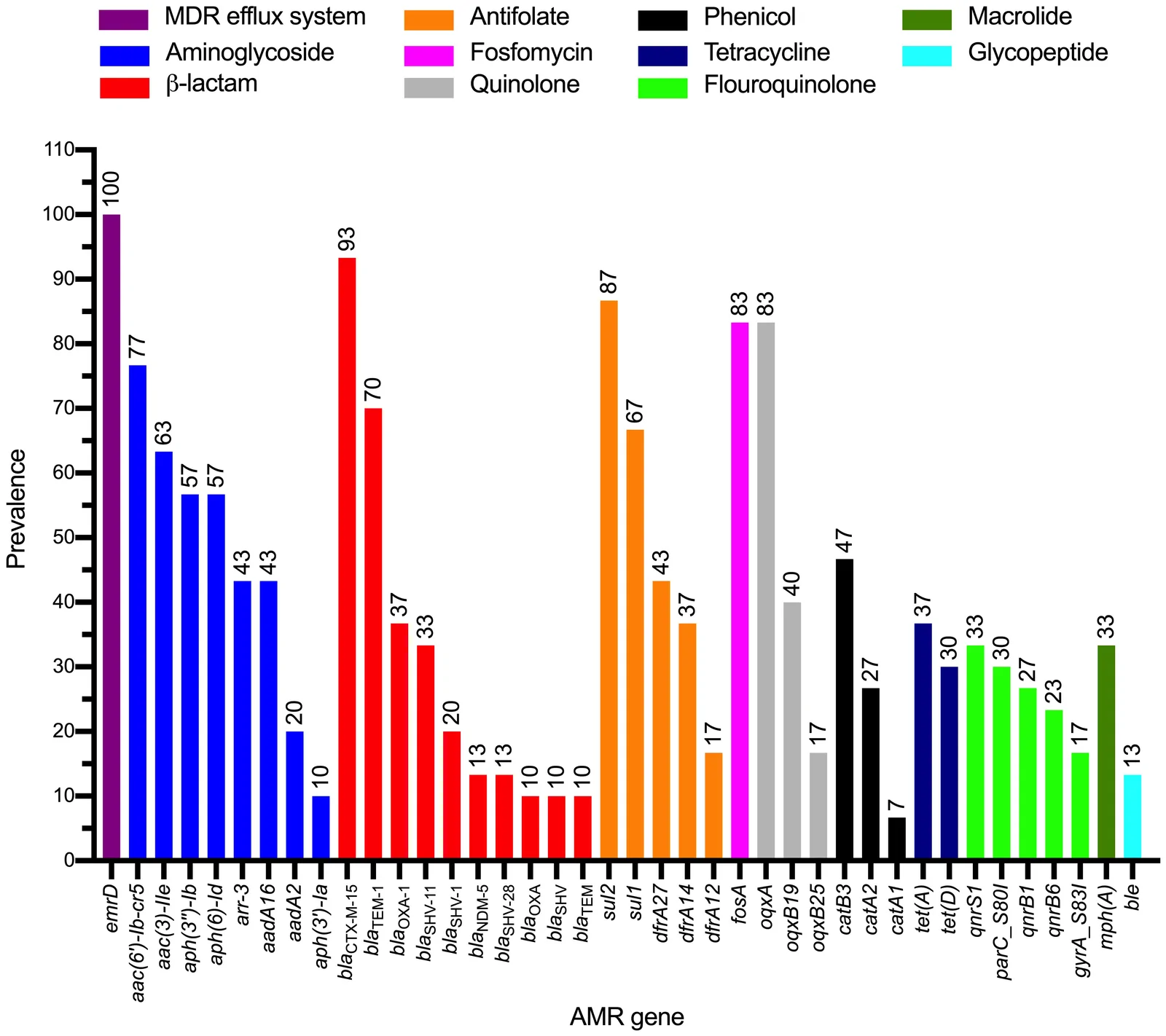
Prevalence of each AMR gene among the 30 *Klebsiella* isolates in this study per antibiotic class.

### Genetic determinants of MDR and XDR

Hierarchical clustering of *K. pneumoniae* isolates based on combined AST and AMR gene profiles, together with STs and source information, grouped the isolates into five major clusters (Kp1–Kp5), each displaying distinct yet overlapping resistance patterns (**Figure 6**). Kp1 cluster possessed isolates that were susceptible to 7 of the 8 antibiotic classes used in this study and had the fewest ARGs. However, all isolates in the five clusters originated from multiple hospital sources. Across clusters Kp2–Kp5, there was high resistance to cephalosporins (CFZ, FOX, CAZ, CRO, FEP) and widespread presence of diverse β-lactamase genes combinations, but *bla*CTX-M-15, *bla*TEM-1, and *bla*SHV variants were the predominant ESBL genes. The aminoglycoside resistance (GEN, TOB) in Kp3–Kp5 aligned with the detection of aminoglycoside-modifying enzyme genes such as *aac(3)-IIe*, *aac(6′)-Ib-cr*, *aadA* variants and *aph* variants. Carbapenem non-susceptibility varied across the Kp3 and Kp5 clusters, with 3 out of 4 isolates (ZLU00442384, ZLU0042378, and ZLU0042365) harbouring *bla*NDM-5 resistant to ETP and MEM. Two isolates (ZLU042385 in Kp3 and ZLU0042367 in Kp5) lacked the *bla*NDM-5 gene but were resistant to both ETP and MEM. Resistance to trimethoprim–sulfamethoxazole, tetracycline, and fosfomycin was explained by the detection of *dfrA/sul* genes in Kp2–Kp5 (**Figure 6**). STs with multiple isolates (ST17, ST377, ST570, ST607 and ST831) clustered together suggesting shared AMR profiles and genotypes, while the presence of multiple STs within clusters may indicate horizontal gene transfer (HGT). Examining the distribution of these clusters by hospital origin (**Supplementary Figure S1**) reveals that while UTH hosts isolates from all five clusters (Kp1–Kp5), NUTH and LUTH predominantly harbour isolates from clusters Kp2–Kp4, reinforcing the notion of a shared regional pool of circulating MDR lineages. Of the seven XDR *K. pneumoniae* isolates (ZLU0042384, ZLU0042378, ZLU0042385, ZLU0042365, ZLU0040288, ZLU0042367 and ZLU0042376), only ZLU0040288 originated from NUTH patients and the rest from UTH. Further, among the seven XDR isolates, five were clinically dominant clones (ST101, ST307, ST377 (*n* = 2), and ST607), that have previously been reported as XDR (Salvador-Oke et al., 2024; Idrees et al., 2025; Olwagen et al., 2025). The other two were ST13 and ST831, which have not yet been reported to have an XDR profile.

**Figure 6:**
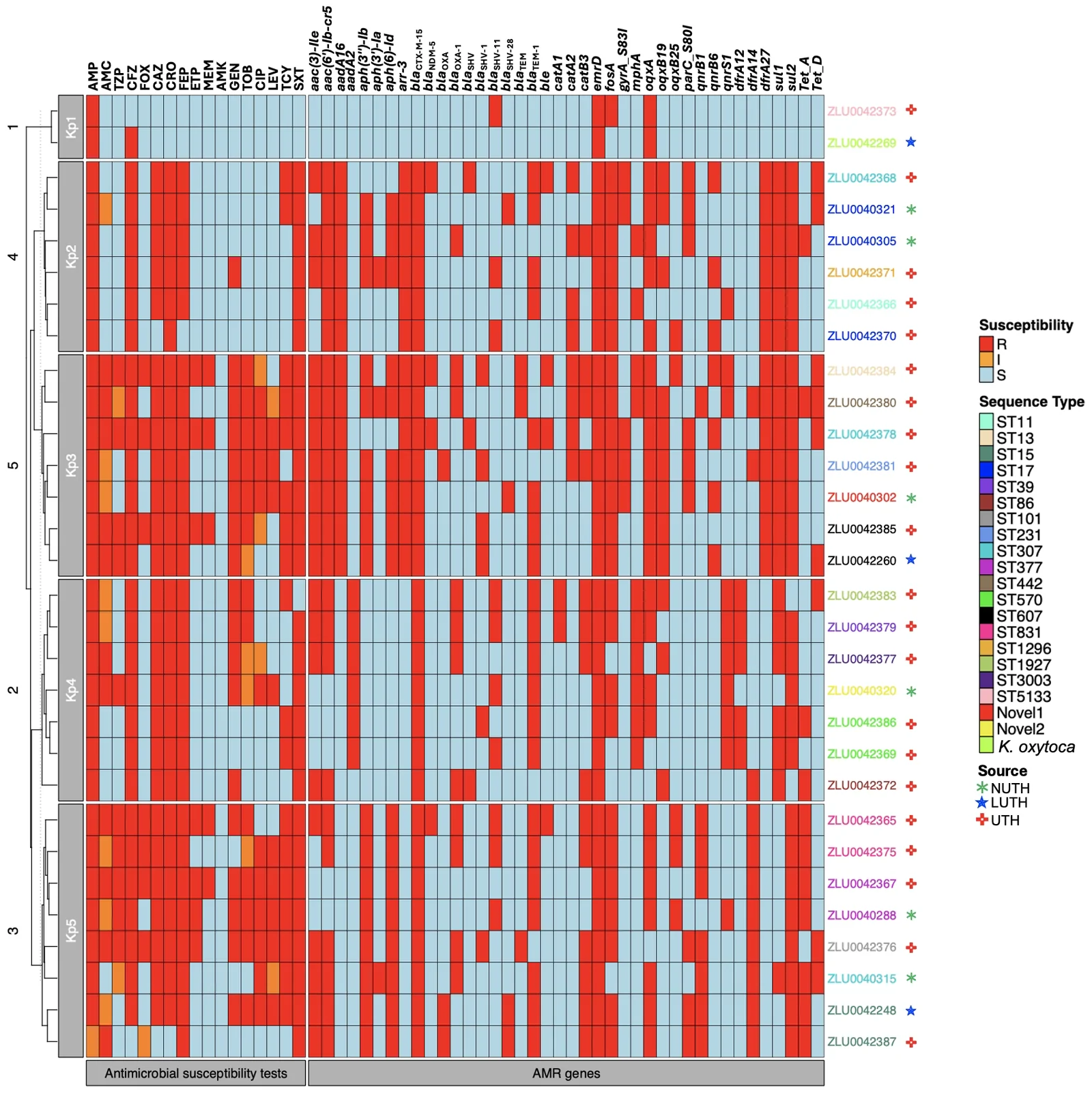
Hierarchical clustering of isolates based on AST profiles and AMR gene content. The heatmap shows phenotypic susceptibility to 18 antibiotics and the presence of ARGs, with corresponding STs and isolate sources indicated. Five major clusters (Kp1–Kp5) were identified. AMP, ampicillin; AMC, amoxicillin–clavulanic acid; TZP, piperacillin–tazobactam; CFZ, cefazolin; FOX, cefoxitin; CAZ, ceftazidime; CRO, ceftriaxone; FEP, cefepime; ETP, ertapenem; MEM, meropenem; AMK, amikacin; GEN, gentamicin; TOB, tobramycin; CIP, ciprofloxacin; LEV, levofloxacin; TCY, tetracycline; SXT, trimethoprim-sulfamethoxazole.

### Virulence-associated gene profiles

Known virulence factors involved in adherence, biofilm formation, capsule synthesis regulation, immune evasion, secretion system, serum resistance, siderophore expression (enterobactin, yersiniabactin, aerobactin, and salmochelin), allantoin utilization, and enterotoxin generation (Choby et al., 2020) were detected among the isolates. All isolates possessed ECP genes (adherence and biofilm formation), *ompA* (immune evasion), *ent* (enterobactin) and *fepA–G* (enterobactin). Others that appeared predominantly were *fim* (adherence and biofilm formation), *ykgK* (adherence) and secretion system encoding (*xcpA/pilD* and *xcpR*) (**Supplementary Table S2**). Virulence profiling identified ZLU0042372 (ST86) as the isolate with the most prominent hypervirulence-associated genotype (**Figure 7**; **Supplementary Table S2**). It carried the complete aerobactin locus (*iucA-D* and *iutA*) and complete salmochelin locus (*iroB-D* and *iroN*) (**Supplementary Table S2**), representing the characteristic combination of siderophore systems strongly associated with hypervirulent *K. pneumoniae*. In contrast, complete yersiniabactin (*ybtA-X*) was detected in 18 isolates spanning diverse sequence types, including ST11, ST15, ST17, ST101, ST307, ST377, ST231 and ST607, but these isolates lacked the aerobactin and salmochelin loci. Notably, ST307 was represented by three isolates (ZLU0040315, ZLU0042368 and ZLU0042378), all carrying complete yersiniabactin-associated genes, while ST15 and ST607 each showed similar yersiniabactin profiles. The hypervirulence-associated markers *rmpA*, *clb*, *peg* - 344 and *wz* (Choby et al., 2020) were absent from all 30 isolates. Collectively, these findings identify ZLU0042372 (ST86) as the principal candidate hypervirulent isolate, based on its combined aerobactin and salmochelin profile. However, definitive phenotypic classification as hypervirulent would require complementary phenotypic or functional validation.

**Figure 7:**
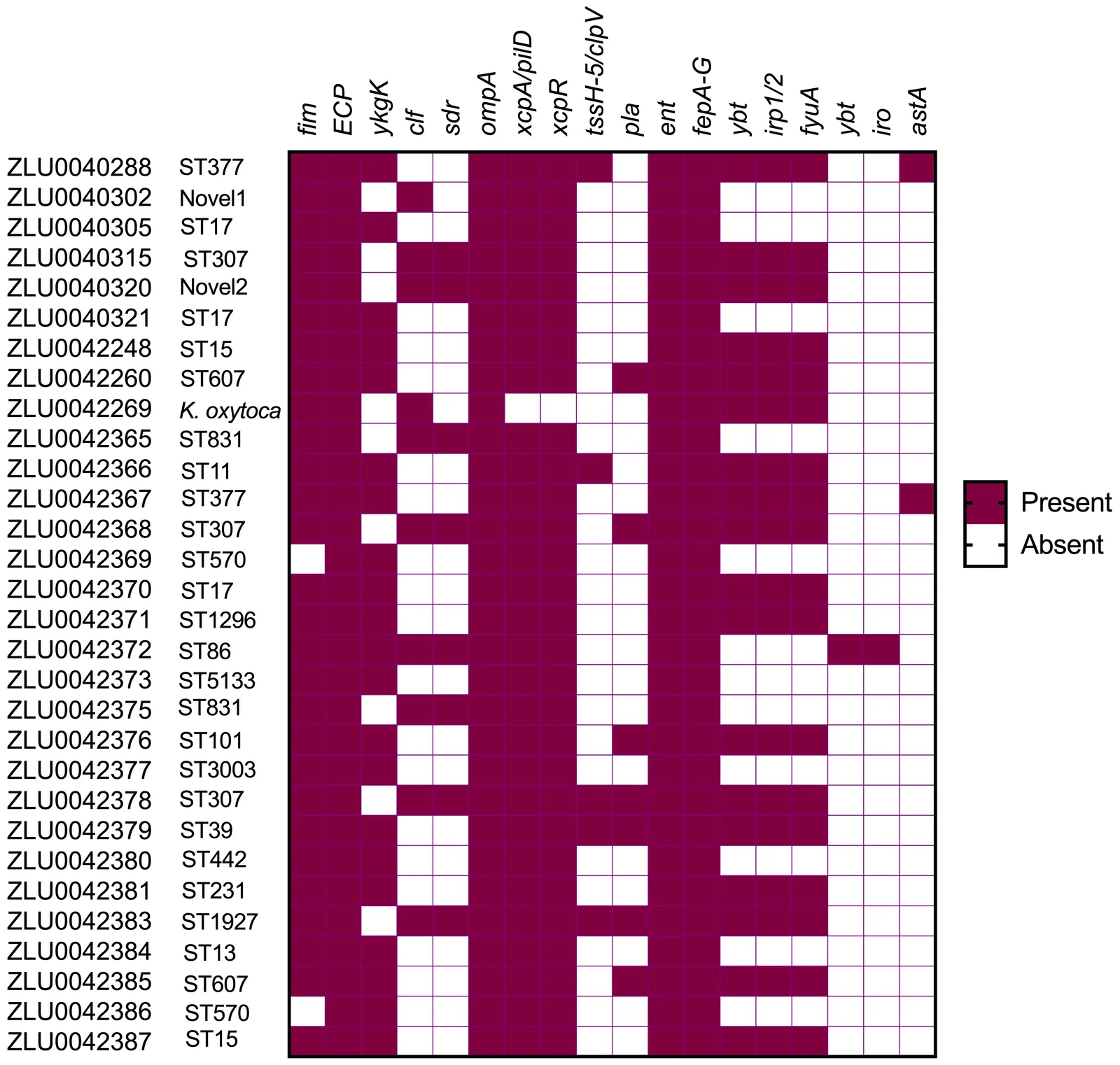
Virulence-associated gene profiles among the 30 *Klebsiella* isolates. Heatmap showing the presence (maroon) or absence (white) of selected virulence-associated genes across the study isolates and their corresponding sequence types (STs). Genes include determinants associated with adherence and biofilm formation (*fim, ECP, ykgK*), adhesion (*clf, sdr*), immune evasion (*ompA*), secretion systems (*xcpA/pilD, xcpR, tssH-5/clpV, pla*), iron acquisition (*ent, fepA-G, ybt, irp1/2, fyuA, iro*) and enterotoxin-associated gene (*astA*).

### Phylogenetic relationships of Zambian *Klebsiella* isolates

The phylogenetic tree shows high diversity among 29 *K. pneumoniae* isolates and 1 *K. oxytoca* isolate (UTH = red, NUTH = green, LUTH = blue) (**Figure 8**). Multiple clades contain isolates from each hospital, with no single hospital-specific lineage, although LUTH and NUTH were underrepresented. For example, UTH isolates (red) appear across the phylogenetic tree, with some clustering tightly together (at the same nodes) and others on separate branches. This polyphyletic pattern is consistent with other studies of *K. pneumoniae*, which also report highly diverse populations across hospital sites. Of note is the node sharing between ZLU0040288 (NUTH) and ZLU0042367 (UTH), both of which are ST377 and share a similar XDR profile and AMR genes. As expected, branch node sharing among the same STs was present. **Figure 8** shows that ST15, ST307, and ST607, represented by ZLU0042248 (LUTH) and ZLU0042387 (UTH); ZLU0040315 (NUTH), ZLU0042368 (UTH) and ZLU0042378 (UTH); ZLU042260 (LUTH) and ZLU0042385 (UTH), respectively, shared the same branch node. Overall, NUTH and LUTH isolates are interspersed among the UTH isolates. Several Zambian isolates cluster with black-coded non-Zambian *K. pneumoniae* strains, indicating shared ancestry with global lineages. Within the *K. pneumoniae* species complex, isolate ZLU0042269 from LUTH clustered together with *K. oxytoca* strains.

**Figure 8:**
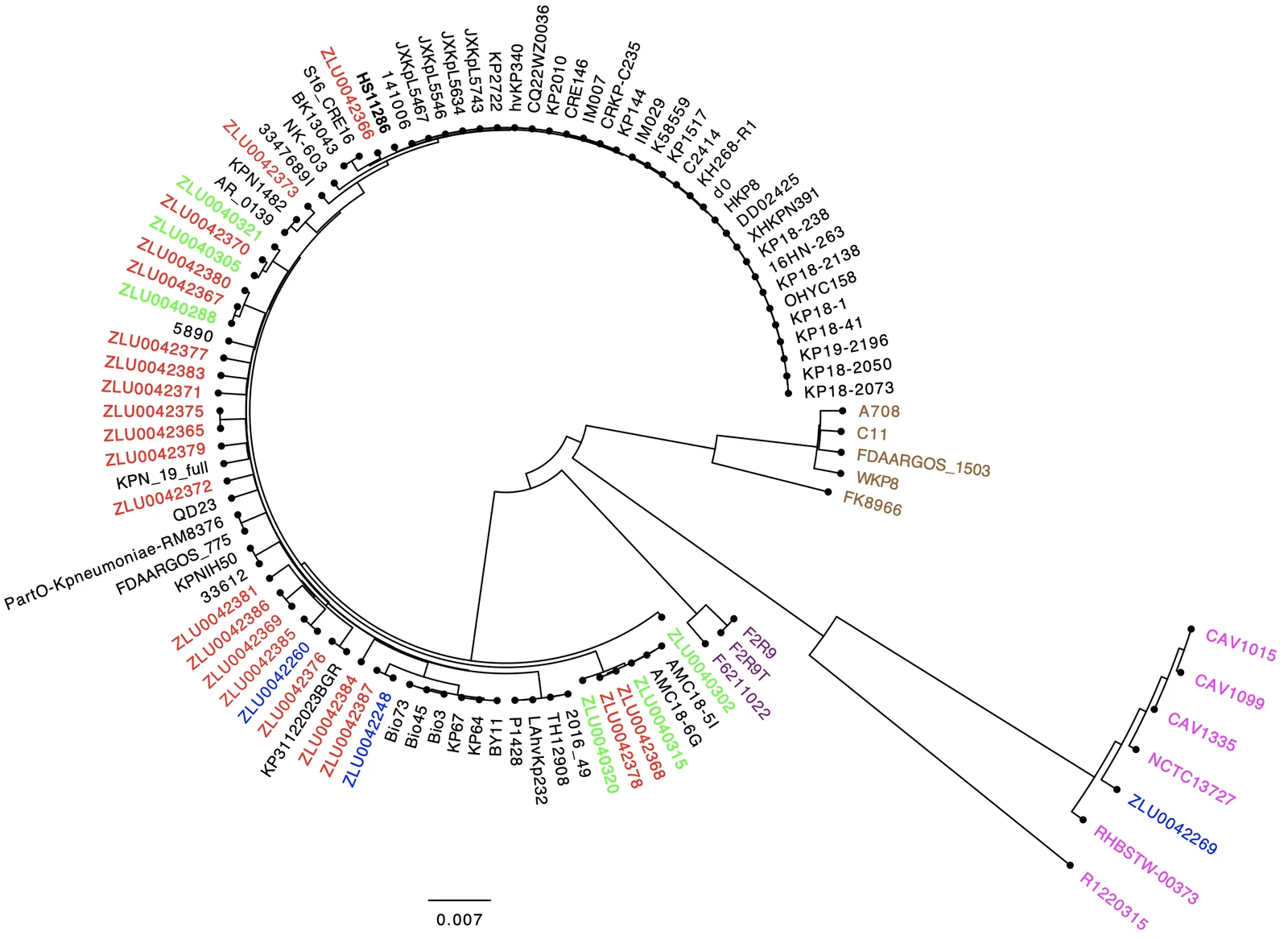
Maximum-likelihood phylogenetic tree of *K. pneumoniae* complex isolates from Zambia and non-Zambian strains. The tree illustrates the phylogenetic relationships among Zambian isolates from UTH (red), NUTH (green), and LUTH (blue), as well as non-Zambian strains. Non-Zambian *K. pneumoniae* strains are shown in black, *K. quasipneumoniae* in brown, *K. variicola* in purple, and *K. oxytoca* in magenta. The *K. pneumoniae* strain HS11286 (bold) was used as the reference genome. Branch lengths represent nucleotide substitutions per site, with the scale bar corresponding to 0.007 substitutions per site.

## Discussion

This multi-site genomic survey represents the first characterisation of *K. pneumoniae* in Zambia. The study provides valuable genomic insights into the molecular epidemiology and determinants of AMR in bloodstream *K. pneumoniae* isolates recovered from inpatients at three tertiary hospitals in Zambia. The findings reveal a high level of genetic diversity, including global high-risk clones ST15, ST307, ST377 and ST607, as well as several minor and previously uncharacterised STs. This diversity underscores the dynamic evolution and dissemination of *K. pneumoniae* lineages across the Zambian healthcare system and situates the local epidemiological picture within the broader sub-Saharan African context, where MDR *K. pneumoniae* has become a major nosocomial threat. The antimicrobial susceptibility profiles of *K. pneumoniae* isolates from NUTH, LUTH, and UTH demonstrated a high burden of AMR across multiple antibiotic classes, with clear patterns of widespread MDR. Only 2/30 isolates were non-MDR.

The detection of 18 different STs, including six dominant lineages (ST11, ST15, ST101, ST307, ST377, and ST607), reflects extensive genomic heterogeneity consistent with a complex hospital transmission landscape. High-risk clones of *K. pneumoniae* ST11, ST15, ST307, ST377, and ST607 are globally disseminated lineages strongly associated with multidrug resistance, particularly carbapenemase production, including KPC, NDM, and OXA-48-like enzymes, contributing to hospital outbreaks and increased mortality in invasive infections (Wyres and Holt, 2018; David et al., 2019; Wyres et al., 2019). The presence of pandemic STs (ST11, ST15, ST101, ST307, and 377), which were strongly associated with MDR, carbapenem resistance, and hospital outbreaks across the three tertiary hospitals, was particularly significant (Peirano et al., 2020; Campos-Madueno et al., 2022; Chatzidimitriou et al., 2025). ST607, by contrast, appears to be regionally established in southern and eastern Africa, where it has been implicated in outbreaks of ESBL-producing *K. pneumoniae* in neonatal units and hospital settings. Only two studies have reported *K. pneumoniae* ST607 in Africa, in Malawi and Kenya. The ST607 has emerged as a major ESBL clone in Malawi and Kenya, associated with IncFIB plasmids and *bla*_CTX-M-15_, and is identical to the Zambian isolates (Lewis et al., 2022; Muraya et al., 2022).

The identification of several minor and novel STs is epidemiologically important as it suggests local diversification and possible emergence of novel lineages under selective antimicrobial pressure. The detection of undesignated STs suggests the presence of potentially novel or uncharacterised lineages. The novel STs could represent cryptic reservoirs that may acquire resistance or virulence genes through horizontal gene transfer, a phenomenon well documented in Enterobacterales. Further, the detection of an ST, particularly ST15, ST307, ST377 and ST607, in multiple tertiary hospitals may indicate transmission through patient referral networks, community transmission or regional dissemination, underscoring the need for integrated genomic surveillance across healthcare facilities. The distribution of resistance clusters (Kp1-Kp5) across the three hospitals further supports this observation. While UTH, as the national referral hospital, exhibited the full range of resistance clusters (Kp1-Kp5), NUTH and LUTH predominantly harboured isolates from clusters Kp2-Kp4 (**Supplementary Figure S1**). This pattern suggests that UTH may serve as a central hub for the accumulation and dissemination of diverse MDR lineages, which subsequently seed regional hospitals through patient referrals and inter-facility transfers. The absence of the most resistant cluster (Kp5) from LUTH and its limited representation at NUTH may reflect differences in patient populations, antimicrobial prescribing practices, or infection control capacities across sites. Alternatively, this could be an artefact of the smaller sample sizes from these hospitals, underscoring the need for expanded surveillance to confirm these preliminary observations. Nevertheless, the shared presence of clusters Kp2-Kp4 across all three sites indicates that regional dissemination of MDR *K. pneumoniae* is already occurring and warrants coordinated, multi-facility infection prevention and control interventions.

The dominance of the IncFIB(K)_1_Kpn3 plasmid replicon, detected in 86% of isolates across multiple STs, is striking. This replicon, previously described in association with ESBL and carbapenemase genes, is a well-recognised driver of AMR dissemination within *K. pneumoniae* (Martínez-Álvarez et al., 2025; Sun et al., 2025). Its widespread distribution across phylogenetically distinct lineages suggests that plasmid-mediated gene flow plays a critical role in maintaining the MDR phenotype in these hospital environments. The frequent co-carriage of multiple replicons in the same isolate, especially IncFIB, IncFII, and IncR, provides further evidence of extensive plasmid plasticity and recombination potential. This is consistent with findings from other SSA countries, such as Kenya, Malawi, and South Africa, where IncF-type plasmids dominate among hospital-associated *K. pneumoniae* and contribute to the rapid spread of *bla*_CTX-M-15_ and *bla*_OXA-48_ (Madni et al., 2021; Lewis et al., 2022; Muraya et al., 2022; Hetsa et al., 2025).

The presence of the InFIB(K)_1_Kpn3 replicon in both dominant high-risk clones and minor STs (**Figure 3B**) illustrates the versatility of these mobile genetic elements in crossing clonal boundaries. Such plasmid promiscuity enhances the potential for rapid adaptation and complicates infection control efforts, as resistance can persist even if a dominant clone is eliminated. Further, the detection of ST39, ST86, ST101, ST5133, and ST831 co-harbouring IncFIB(K)_1_Kpn3 and IncHI1B_1_pNDM-MAR replicons suggests convergence of high-risk or emerging clones with NDM-associated MDR plasmids, enhancing the potential for horizontal gene transfer and limiting therapeutic options. The identification of co-resident Col-type plasmids, often associated with quinolone and fosfomycin resistance, further broadens the repertoire of resistance.

All isolates in this study carried multiple ARGs, confirming the widespread MDR observed. The predominance of β-lactamase genes, particularly *bla*_CTX-M-15_ (93%), *bla*_SHV_ variants (78%), and *bla*_TEM_ variants (75%), reflects the continuing dominance of ESBL-mediated resistance in Zambian *Klebsiella* populations. The accumulation of aminoglycoside-modifying enzyme genes, such as *aac(6’)-Ib-cr5* and *aph(3’)-Ib*, together with sulfonamide (*dfrA*, *sul1*, *sul2*), fosfomycin (*fosA*), and quinolone (*oqxA* and *oqxB19*) resistance determinants, indicates the widespread circulation of composite resistance plasmids.

Particularly notable was the observation that ST13, ST231, ST307, and ST442 harboured the largest number of ARGs (21–25) (**Figure 4**). For the high-risk MDR clones, ST231 and ST307, this finding is expected, however, ST13 and ST442 are somewhat high but still plausible, especially since they carried multiple plasmid replicons. ST13 and ST442 are not globally disseminated clones but are clinically relevant healthcare-associated lineages and increasingly being reported to carry ESBL and carbapenemase genes, contributing to MDR infections (Marcade et al., 2013; Martins et al., 2022). The hypervirulent ST307 has been implicated in neonatal and intensive care unit outbreaks in sub-Saharan Africa, where it often carries *bla*_OXA-181_ and *bla*_CTX-M-15_ (Lowe et al., 2019; Salvador-Oke et al., 2024; Kidenya et al., 2025; Olwagen et al., 2025). The detection of two MDR and one XDR isolates in this study, each possessing the carbapenemase resistance determinant (*bla*_NDM-5_), reinforces their relevance as an emerging high-risk lineage in the region. From a public health standpoint, the circulation of multiple high-risk clones across major referral hospitals poses a serious infection-control challenge. Conversely, the detection of diverse STs with MDR and XDR profiles within UTH suggests that tertiary hospitals act as amplification hubs for both endemic and imported MDR lineages. Without effective screening, environmental decontamination, and AMS, these clones could disseminate to secondary hospitals and community settings.

The virulence profiles demonstrate that Zambian *Klebsiella* isolates harbour diverse determinants associated with adherence, biofilm formation, immune interaction, secretion and iron acquisition typical of bloodborne *Klebsiella* isolates. The widespread detection of *fim*, ECP-associated genes, y*kgK*, *ompA*, and enterobactin genes suggests that core colonisation, persistence and iron-acquisition mechanisms, which may facilitate survival in healthcare environments, were broadly distributed. Yersiniabactin was detected across several sequence types, including high-risk MDR lineages. Notably, ST86 isolate ZLU0042372 carried complete aerobactin and salmochelin loci, identifying it as a candidate hypervirulent isolate. However, the absence of *rmpA, clb, peg* - 344 and *wz* across all isolates indicates that a broader hypervirulence genotype was uncommon. Phylogenetic analysis demonstrated substantial genetic diversity among the Zambian *Klebsiella* isolates, with isolates from UTH, NUTH and LUTH distributed across multiple clusters rather than forming distinct hospital-specific lineages. Sequence types represented by more than one isolate shared the same branch node, although their source, AMR and ARG profiles were differed. However, ZLU0040288 (NUTH) and ZLU0042367 (UTH) both ST377 and shared similar XDR and ARG profiles and virulence genes. The highly similar phenotypic and genomic characteristics of ZLU0040288 and ZLU0042367 suggest that the two isolates are the same clone despite being isolated from different hospitals. Overall, several Zambian isolates clustered with non-Zambian *K. pneumoniae* strains, indicating that local isolates occur within broader genetic backgrounds. This pattern is consistent with the 18 MLST-defined sequence types and the presence of multiple high-risk lineages. However, phylogenetic relatedness alone does not establish direct transmission, particularly given the cross-sectional design and smaller sample sizes from NUTH and LUTH. The clustering of one LUTH isolate with *K. oxytoca* further demonstrates the value of genomic species confirmation.

Altogether, this study reveals an alarming scenario in which Zambian hospitals harbour diverse MDR and XDR *K. pneumoniae* lineages dominated by globally recognised high-risk clones carrying multiple resistance determinants and virulence genes. The patterns observed closely align with current AMR dynamics across sub-Saharan Africa (Lowe et al., 2019; Kidenya et al., 2025; Olwagen et al., 2025) characterised by plasmid-driven dissemination of ESBLs and carbapenemases (Silago and Mshana, 2022; Byarugaba et al., 2024; Mills et al., 2024; Olaitan et al., 2025). The findings highlight the urgent need for coordinated genomic surveillance, strict IPC practices, and rational AMU policies to mitigate the spread of these MDR pathogens.

### Limitations

Future studies integrating clinical outcomes, virulence profiling, and environmental sampling will be critical for understanding transmission dynamics and informing targeted interventions. The use of short-read sequencing data limited our ability to resolve the genomic locations of ARGs and plasmid replicons accurately. In particular, Illumina sequencing alone cannot reliably distinguish whether ARGs and replicons are located on plasmids or integrated into the chromosome, nor can it determine whether the presence of multiple replicon types reflects multiple distinct plasmids or single multi-replicon plasmids. Further, a relatively small sample size constrained our ability to comprehensively assess the genetic diversity of *K. pneumoniae* across the study sites. This limitation was especially evident in Livingstone and Ndola, from which only 3 and 6 isolates were available, respectively.

Finally, the analysis was restricted to bloodstream isolates, which may bias the findings toward strains adapted to invasive disease and limit the generalisability of the results to *K. pneumoniae* populations circulating in other clinical niches or environmental reservoirs.

## Data Availability

The datasets presented in this study are deposited in the NCBI repository, accession number PRJNA1514012.
