# Supplementary material for: Genomic insights into multidrug and extensively drug-resistant Klebsiella pneumoniae from bloodstream infections at three Zambian tertiary hospitals
Source: Front Antibiot. 2026 Oct 9;5:1933211. doi: 10.3389/frabi.2026.1933211 (PMC13690789; doi:10.3389/frabi.2026.1933211)
Supplement: Supplementary file 1 [file Image1.pdf]

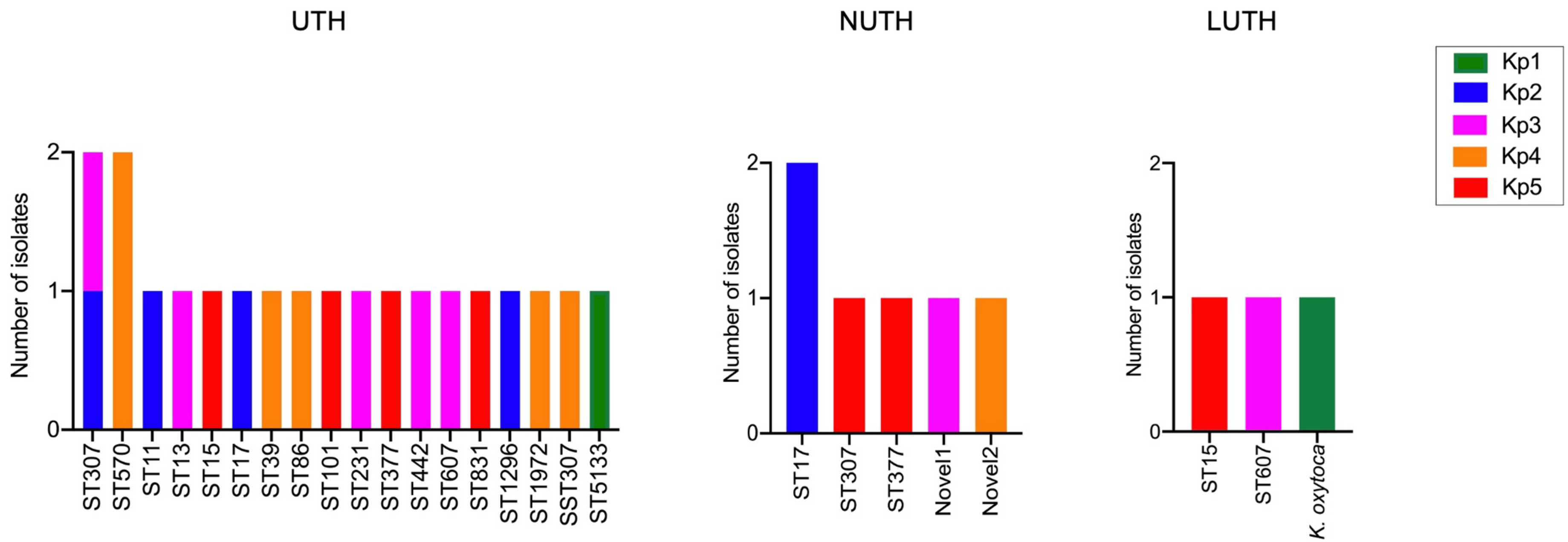

Figure S1: Distribution of *K. pneumoniae* sequence types among 30 isolates from UTH, NUTH and LUTH. Bar height represents the number of isolates, while colours indicate phylogenetic cluster membership (Kp1–Kp5).
