# Supplementary material for: Genomic insights into multidrug and extensively drug-resistant Klebsiella pneumoniae from bloodstream infections at three Zambian tertiary hospitals
Source: Front Antibiot. 2026 Oct 9;5:1933211. doi: 10.3389/frabi.2026.1933211 (PMC13690789; doi:10.3389/frabi.2026.1933211)
Supplement: Supplementary file 2 [file Table1.docx]

Table S1: Isolate sequence typing metadata

| **Isolate ID** | **Source** | **Species** | **Sequence Type** | **Allelic Profile** | | | | | | |
| --- | --- | --- | --- | --- | --- | --- | --- | --- | --- | --- |
| ZLU0040288 | NUTH | *Klebsiella pneumoniae* | 377 | gapA(10) | infB(20) | mdh(2) | pgi(1) | phoE(9) | rpoB(11) | tonB(12) |
| ZLU0040302 | NUTH | *Klebsiella pneumoniae* | Novel1 | gapA(326?) | infB(256?) | mdh(2) | pgi(399?) | phoE(13) | rpoB(1) | tonB(16) |
| ZLU0040305 | NUTH | *Klebsiella pneumoniae* | 17 | gapA(2) | infB(1) | mdh(1) | pgi(1) | phoE(4) | rpoB(4) | tonB(4) |
| ZLU0040315 | NUTH | *Klebsiella pneumoniae* | 307 | gapA(4) | infB(1) | mdh(2) | pgi(52) | phoE(1) | rpoB(1) | tonB(7) |
| ZLU0040320 | NUTH | *Klebsiella pneumoniae* | Novel2 | gapA(4) | infB(1) | mdh(2) | pgi(52) | phoE(1) | rpoB(173) | tonB(7) |
| ZLU0040321 | NUTH | *Klebsiella pneumoniae* | 17 | gapA(2) | infB(1) | mdh(1) | pgi(1) | phoE(4) | rpoB(4) | tonB(4) |
| ZLU0042248 | LUTH | *Klebsiella pneumoniae* | 15 | gapA(1) | infB(1) | mdh(1) | pgi(1) | phoE(1) | rpoB(1) | tonB(1) |
| ZLU0042260 | LUTH | *Klebsiella pneumoniae* | 607 | gapA(2) | infB(1) | mdh(2) | pgi(80) | phoE(10) | rpoB(1) | tonB(155) |
| ZLU0042365 | UTH | *Klebsiella pneumoniae* | 831 | gapA(2) | infB(1) | mdh(1) | pgi(1) | phoE(10) | rpoB(1) | tonB(18) |
| ZLU0042366 | UTH | *Klebsiella pneumoniae* | 11 | gapA(3) | infB(3) | mdh(1) | pgi(1) | phoE(1) | rpoB(1) | tonB(4) |
| ZLU0042367 | UTH | *Klebsiella pneumoniae* | 377 | gapA(10) | infB(20) | mdh(2) | pgi(1) | phoE(9) | rpoB(11) | tonB(12) |
| ZLU0042368 | UTH | *Klebsiella pneumoniae* | 307 | gapA(4) | infB(1) | mdh(2) | pgi(52) | phoE(1) | rpoB(1) | tonB(7) |
| ZLU0042369 | UTH | *Klebsiella pneumoniae* | 570 | gapA(2) | infB(1) | mdh(2) | pgi(37) | phoE(8) | rpoB(1) | tonB(15) |
| ZLU0042370 | UTH | *Klebsiella pneumoniae* | 17 | gapA(2) | infB(1) | mdh(1) | pgi(1) | phoE(4) | rpoB(4) | tonB(4) |
| ZLU0042371 | UTH | *Klebsiella pneumoniae* | 1296 | gapA(4) | infB(1) | mdh(2) | pgi(1) | phoE(7) | rpoB(70) | tonB(12) |
| ZLU0042372 | UTH | *Klebsiella pneumoniae* | 86 | gapA(9) | infB(4) | mdh(2) | pgi(1) | phoE(1) | rpoB(1) | tonB(27) |
| ZLU0042373 | UTH | *Klebsiella pneumoniae* | 5133 | gapA(2) | infB(9) | mdh(1) | pgi(20) | phoE(13) | rpoB(4) | tonB(16) |
| ZLU0042375 | UTH | *Klebsiella pneumoniae* | 831 | gapA(2) | infB(1) | mdh(1) | pgi(1) | phoE(10) | rpoB(1) | tonB(18) |
| ZLU0042376 | UTH | *Klebsiella pneumoniae* | 101 | gapA(2) | infB(6) | mdh(1) | pgi(5) | phoE(4) | rpoB(1) | tonB(6) |
| ZLU0042377 | UTH | *Klebsiella pneumoniae* | 3003 | gapA(4) | infB(1) | mdh(1) | pgi(1) | phoE(313) | rpoB(10) | tonB(9) |
| ZLU0042378 | UTH | *Klebsiella pneumoniae* | 307 | gapA(4) | infB(1) | mdh(2) | pgi(52) | phoE(1) | rpoB(1) | tonB(7) |
| ZLU0042379 | UTH | *Klebsiella pneumoniae* | 39 | gapA(2) | infB(1) | mdh(2) | pgi(4) | phoE(9) | rpoB(1) | tonB(14) |
| ZLU0042380 | UTH | *Klebsiella pneumoniae* | 442 | gapA(10) | infB(20) | mdh(2) | pgi(1) | phoE(9) | rpoB(11) | tonB(14) |
| ZLU0042381 | UTH | *Klebsiella pneumoniae* | 231 | gapA(2) | infB(6) | mdh(1) | pgi(3) | phoE(26) | rpoB(1) | tonB(77) |
| ZLU0042383 | UTH | *Klebsiella pneumoniae* | 1927 | gapA(2) | infB(1) | mdh(171) | pgi(1) | phoE(10) | rpoB(4) | tonB(13) |
| ZLU0042384 | UTH | *Klebsiella pneumoniae* | 13 | gapA(2) | infB(3) | mdh(1) | pgi(1) | phoE(10) | rpoB(1) | tonB(19) |
| ZLU0042385 | UTH | *Klebsiella pneumoniae* | 607 | gapA(2) | infB(1) | mdh(2) | pgi(80) | phoE(10) | rpoB(1) | tonB(155) |
| ZLU0042386 | UTH | *Klebsiella pneumoniae* | 570 | gapA(2) | infB(1) | mdh(2) | pgi(37) | phoE(8) | rpoB(1) | tonB(15) |
| ZLU0042387 | UTH | *Klebsiella pneumoniae* | 15 | gapA(1) | infB(1) | mdh(1) | pgi(1) | phoE(1) | rpoB(1) | tonB(1) |

NUTH – Ndola University Teaching Hospital

LUTH – Livingstone University Teaching Hospital

UTH – University Teaching Hospital
